# Surgery

**Published:** 1906-09-08

**Authors:** 


### Progress in Medicine and Surgery.

### SURGERY.

**On the Possibility of Operative Interference in Injuries and Disease of the Heart.**—The number of cases of wounded heart which have been successfully sutured is steadily increasing, and it is therefore worth while to consider what are the essential difficulties of cardiac surgery, how they may be overcome, and the possibility that there is of cardiac disease as well as injury ultimately becoming a surgical province. At the outset it must be admitted that however our methods of treatment may improve, the majority of patients who have received serious wounds of the heart will die before any treatment can be adopted. But dealing with the minority of cases in which life is prolonged for a sufficient time for surgical treatment to be adopted, we are then confronted with four main difficulties. First there is the danger of any exposure or manipulation of the heart bringing it to a standstill and so causing death. But whilst this is the most obvious danger, it appears from the record of published cases not to be a very real one. The heart can be seized between the fingers and thumb when the suturing is being carried out, without causing it to stop beating. And, indeed, the mere manipulation of the heart or direct cardiac massage has in at least one case restored the circulation when it had come to a standstill under the influence of an anæsthetic. The causes of death in wound of the heart are hæmorrhage and pressure on the heart by blood in the pericardium. And it is evident, therefore, that operative interference will in every case increase the prospects of life where life is possible, and not diminish them under hopeless conditions. The next danger which proves fatal in most cases is the mechanical interference with the heart-action by blood in the pericardium. And this being so is the great reason to immediate operation in all cases of wounds over the cardiac region accompanied by an immediate increase of cardiac dullness. The mere relief of pericardial pressure and the turning out of blood-clots will save life in these cases, the suture of the small wound in the heart is a comparatively small matter, and in one case reported by Picqué, even this was not necessary, as the bullet wounds had closed spontaneously. The third danger, which especially attends stabs, is septic infection of the pericardium and pleura. And as this is introduced at the time of the injury, no aseptic precautions in operating will avoid it. In a case operated on by de Fourmestraux a stab wound of the left ventricle was sewn up, but both pericardium and pleura were invaded by septic inflammation, in spite of which the patient ultimately recovered. Here again it is obvious that the danger will increase with delay. And lastly there is the danger of injury to the left pleura and lung. Even in the normal chest any wound above the level of the fourth costal cartilage must involve these structures, and if any emphysema exists, they may cover the whole pericardial area. When such an injury has complicated a wound over the heart, its presence will be indicated by signs of fluid in the left pleura. This will, of course, greatly add to the gravity of the case, but need not prevent operation being undertaken. In one case it was found that though there had been a penetrating wound through pleura, lung, and pericardium to the heart, yet the chief hæmorrhage proceeded from the last-named structure and recovery took place after this had been sewn up. The vessels which may be divided in the anterior edge of the lung are only small, and bleeding from them will probably stop spontaneously; so that the chief importance of a wound of the left pleura complicating that of the pericardium and heart will be the free space thereby opened up for bleeding and subsequent septic infection. Now if an injury of the heart-wall, always complicated by an external wound and a wound of the pericardium, and often by a wound of the lung and pleura can be successfully cured by surgical means, the question arises whether it may be possible to deal directly with any form of valvular disease of the heart. Some years ago Sir Lauder Brunton urged that surgeons should consider the possibility of relieving mitral stenosis. As a result of his experiments upon animals, he thought this could be done by manipulating through the carotid in the neck. But it would be much easier and more definite to do this through the wall of the left ventricle after exposing the heart through the pericardium. The mere exposure of the heart and the existence of a comparatively large wound in its substance having been proved to be well borne, there seems to be no reason why a needle-knife could not be introduced into the ventricle and made to engage in the mitral orifice which then could be incised in several directions. At any rate there seems to be nothing more impossible about this procedure to-day than there was in operating on the abdominal viscera 50 years ago.

**On the Causes and Treatment of some Varieties of Sprained Knee.**—If the importance of an injury be in any way proportionate to the commonness of its occurrence and to the difficulty of its treatment, then a sprained knee is one of the most important surgical conditions that the practitioner will ever have to deal with. Sprains, fractures, and dislocations are all injuries which every practitioner is expected by the public to be able to diagnose and treat without any assistance from a specialist. And yet they all at times afford the most difficult problems for solution, and any failure will be constantly carried about the world by a complaining patient as evidence of the practitioner's want of skill. The liability of the knee-joint to suffer permanently as the result of a slight injury is due partly to its complicated structure and partly to the constant strain to which it is submitted in carrying the body-weight in biped progression. In considering the condition of "sprain" in a knee we have in the first place to carefully exclude those cases of injury or disease outside the knee in which pain may be referred to the joint. It is only necessary to mention such conditions as tubercular hip or popliteal aneurism, to indicate examples of these diseases. In the second place it is very important to remember that organic disease of the joint may be started by a sprain or that some latent disease may be revealed for the first time as the result of a slight injury. A tubercular knee, or even osteo-myelitis of the lower end of the femur or osteo-arthritis may all have this relation to a "sprain." But when extra-articular disease and intra-articular disease have been excluded, there remain the large class of sprained knees which include all cases of ligamentous and internal derangements of the joint. An injury which is generally of the nature of a strain or a twist is followed by pain, swelling and stiffness of the joint, which may be fixed in a position of flexion or extension. The symptoms subside under rest and palliative treatment by pressure or counter-irritants, but re-appear at a longer or shorter interval directly the limb is used. The recurrent attacks are either repetitions of the first attack or else a constant sense of pain and weakness, with some swelling of the joint. And the difficult problem must be solved sooner or later as to whether more rest is wanted, or more exercise, or an operation, or can nothing be done and must the patient go about with a "chronic" knee. If the case presents such typical signs of a displaced cartilage or of a loose body that a definite diagnosis is possible, then there can be no doubt about the necessity for surgical interference. The most definite of these signs is the locking of the joint, which is only momentary in the case of a loose body, but is more permanent and often has to be "reduced" by the patient or surgeon in the case of a displaced semi-lunar cartilage. Further, the loose body may be felt under the skin and can be moved about. The only exception that can be made to the rule of operating on all these cases of "typical internal derangements" is in the case of a displaced cartilage seen directly after the original injury. In this case the limb should be kept immobilised absolutely for three to four weeks whilst the patient lies up for two to three weeks. This will give an opportunity for the torn ligament to unite and the cartilage will then be almost as firmly fixed as before the accident. But if once the stage of "recurrent synovitis," that is, of second or more attacks of pain and stiffness is reached, then there is nothing that will cure the condition except opening the joint and removing the injured cartilage. But then there are many cases which are not at all typical of either a loose body or a displaced cartilage. These atypical cases resemble the others in a general way, but there is an absence of the definite "locking" of the joint and of a palpable loose body. There can be no doubt about the right treatment of these cases in the first instance. In any case the limb must be fixed on to a back splint and a recumbent position maintained until signs of heat and tenderness have subsided. If the effusion has not all gone in one week, firm pressure by bandaging should be added to the splint treatment. And this will be sufficient to cure many cases. But in others the fluid either persists or more often returns after a longer or shorter interval, accompanied by pain and stiffness. It has been pointed out lately by Barker in England and Flint in America, that this condition is often due to injury with subsequent hypertrophy of the synovial fringes in the joint which are known as the ligamenta mucosa and alaria. If the joint be opened in these cases it is found to be filled with glairy mucoid fluid often containing blood and fibrin. The synovial fringes at the side of the patella which can be exhibited by flexing the knee and rotating the leg outwards, are thickened and present long villous processes. It is probably the catching of these tags between the joint surfaces that gives rise to the pain, bleeding, and recurrent synovitis. When they are removed the joint becomes rapidly well and strong. And lastly, there are cases in which traumatic osteo-arthritis has been set up as the result of an injury. It is very difficult to distinguish these cases from those where the synovial fringes are hypertrophied, as in both instances grating and creaking may be felt on movement. If the cartilage has been chipped or cracked and the injured spot forms a centre from which fibrillation and degeneration of the cartilage spreads, then nothing can be done to cure this further than the ordinary palliative treatment of osteo-arthritis. Before opening any knee-joint it is well to remember that any lapse from the strict rectitude of asepsis may lead to disaster. But it is interesting to note that even in cases that do well and heal by first intention, there is generally a rise of the temperature to 102° F. the day following the operation.

